# Safety Profile of TiO_2_-Based Photocatalytic Nanofabrics for Indoor Formaldehyde Degradation

**DOI:** 10.3390/ijms161126055

**Published:** 2015-11-19

**Authors:** Guixin Cui, Yan Xin, Xin Jiang, Mengqi Dong, Junling Li, Peng Wang, Shumei Zhai, Yongchun Dong, Jianbo Jia, Bing Yan

**Affiliations:** 1 China Textile Academy, Jiangnan Branch, Shaoxing 312000, China; cuiguixin3@163.com (G.C.); jiangxin_zfy@163.com (X.J.); lijl0720302003@163.com (J.L.); 2 School of Chemistry and Chemical Engineering, Shandong University, Jinan 250100, China; rizhaoxinyan@126.com (Y.X.); dongmengqi029@163.com (M.D.); smzhai@sdu.edu.cn (S.Z.); jiajianbo03@gmail.com (J.J.); 3 School of Textiles, Tianjin Polytechnic University, Tianjin 300387, China; wangpengtjpu@sina.com (P.W.); teamdong@sina.cn (Y.D.)

**Keywords:** TiO_2_ nanoparticle, formaldehyde degradation, photocatalytic agent, cytotoxicity, nano safety

## Abstract

Anatase TiO_2_ nanoparticles (TNPs) are synthesized using the sol-gel method and loaded onto the surface of polyester-cotton (65/35) fabrics. The nanofabrics degrade formaldehyde at an efficiency of 77% in eight hours with visible light irradiation or 97% with UV light. The loaded TNPs display very little release from nanofabrics (~0.0%) during a standard fastness to rubbing test. Assuming TNPs may fall off nanofabrics during their life cycles, we also examine the possible toxicity of TNPs to human cells. We found that up to a concentration of 220 μg/mL, they do not affect viability of human acute monocytic leukemia cell line THP-1 macrophages and human liver and kidney cells.

## 1. Introduction

Indoor air pollution is a major threat to human health. One potent pollutant is formaldehyde, released by most building materials and household products. Human exposure to formaldehyde through inhalation causes perturbations to immune systems [1] and various cancers. Based on astounding evidence, formaldehyde has been named a carcinogen by both the International Agency for Research on Cancer (June 2004) [2] and the National Toxicology Program (June 2011) [3]. Therefore, air cleaning and formaldehyde degradation are necessities for indoor air quality. With the development of nanotechnology, more and more nanomaterials are being used for efficient air cleaning. For example, In VO_4_ nanoparticles [4], ZnO nanoparticles [5,6,7], titanium dioxide nanoparticles (TNPs) [8] and core shell ZnS/alpha-Fe_2_O_3_ nanoparticles [9] have all been investigated for photocatalytic degradation of formaldehyde in the air. TNPs introduced into the structure of polyester fabrics showed good photocatalytic activity in formaldehyde degradation under UV radiation [10]. Poly(vinyl alcohol) nanofiber webs containing TNPs exhibited a formaldehyde decomposition efficiency of 40% after 2 h, 60% after 4 h, and 80% after 15 h under UV irradiation [11]. Furthermore, doping TNPs with metal ions has proven to be a promising way to further improve the photocatalytic activity of formaldehyde degradation [12]. Our vision for a future solution is to incorporate catalytic nanoparticles, such as TNPs, into fabrics used as wall coverings, curtains, and upholstery so that the catalytic degradation of formaldehyde and other organic pollutants occurs naturally and continuously. However, most textiles may lose up to 20% of their mass during their lifetime, so nanoparticles used in production of nanofabrics are at risk of being released into the air and waterways.

Studies [13,14,15] including our own findings [16,17,18,19,20] have shown many examples in which nanomaterials may have adverse impacts on environment and human health. TNPs damage the blood-milk barrier and cause the leakage of nanoparticles into milk and cubs in lactating mice [21]. Effects of TNPs on the viability of human primary peripheral blood mononuclear cells [22], monocyte-derived dendritic cells [22], and primary cultures of human hematopoietic progenitor cells [23] have been reported. Studies on TNP’s interactions with human HaCaT keratinocytes revealed an induction of oxidative stress [24] and a potential toxicity only after long exposure [25]. TNPs also perturb macrophages and induce immune responses [26].

Up to September 2015, there have been 1825 nanotechnology-based consumer products on the market [27]. However, the potential harmful impacts of nanotechnology products are seldom evaluated before their market launch. In this work, we report on formaldehyde degradation by TNP-based nanofabrics and their potential impacts on indoor environments and health. This work reveals critical TNP release from nanofabrics and establishes some standard tests for their effects on the environment and human health.

## 2. Results and Discussion

### 2.1. Characterization of TNP (TiO_2_ Nanoparticle)

The physiochemical properties of nanoparticles play key roles in their catalysis and biological activities. The crystalline form, particle size, zeta potential, and agglomeration of TNPs were characterized.

**Figure 1:**
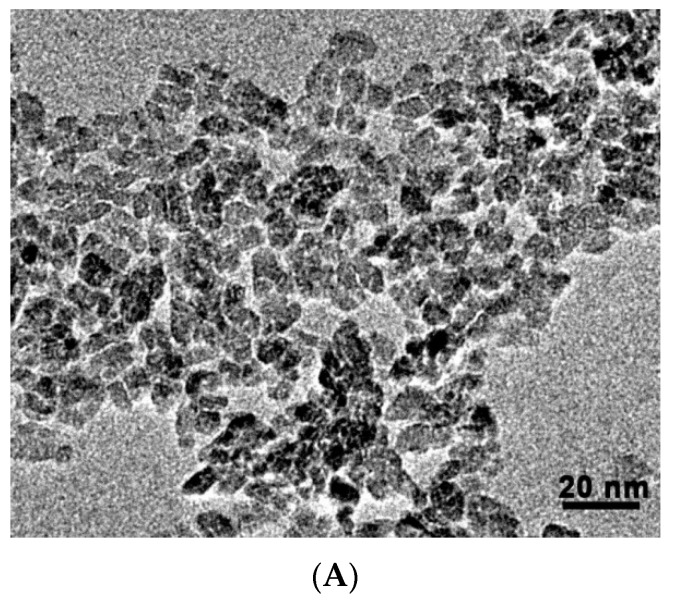

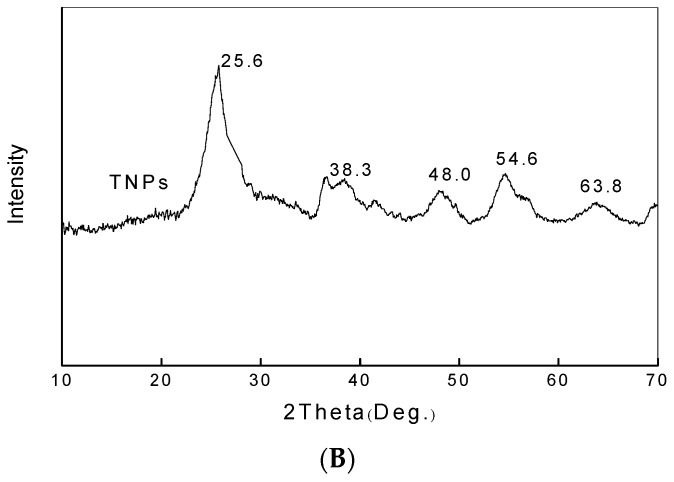
Analysis of the properties of the TNPs. (**A**) TNPs was dispersed by ultrasonication for half an hour and the morphology of the TNPs was observed under a transmission electron microscope; (**B**) The crystalline structure of TNPs was analyzed by X-ray diffraction.

The TEM images of TNP (Figure 1A) showed that their sizes are 7.5 ± 1.5 nm. X-ray diffraction analysis (XRD) analysis demonstrated that the dominant crystalline form of TNPs was anatase form (Figure 1B). In polar solvents, nanoparticles may tend to aggregate. The hydrodynamic diameter of TNP particles in solutions was determined by dynamic light scattering (DLS). Results showed that the dynamic sizes of TNPs are in the range of 30–50 nm in sterile water on the assumption that they were spherical in shape, indicating the degree of TNP aggregation was minor (Table 1). The electrostatic and electrodynamic properties of nanoparticles determine their stability in solution. Such properties can be estimated through the measurement of their zeta potential. Zeta-potential measurement showed that TNPs exhibited fairly positive surface potentials in sterile water, showing that they were relatively stable in aqueous solution (Table 1). In cell culture medium, there are 10% fetal bovine proteins. When proteins are adsorbed onto nanoparticles, the solubility and stability of nanoparticles are improved in general. Next, we analyzed hydrodynamic size and zeta potential of TNPs in the presence of cell culture medium. The hydrodynamic size of TNPs was increased to 97.3 ± 1.5 nm and Zeta potential was reduced to −11.1 ± 0.4 mV (Table 1). Both changes demonstrated that serum proteins were adsorbed onto TNPs in cell cultures.

**Table 1:** The Physicochemical Properties of TNPs.

Physicochemical Property	Value
Diameter by TEM (nm)	7.52 ± 1.5
Hydrodynamic diameter in water (nm)	42.1 ± 7.4
Hydrodynamic diameter in 10% FBS (nm)	97.3 ± 1.5
Zeta potential in water (mV)	+38.9 ± 1.7
Zeta potential in 10% FBS (mV)	−11.1 ± 0.4

### 2.2. TNP-Based Nanofabrics

**Figure 2:**
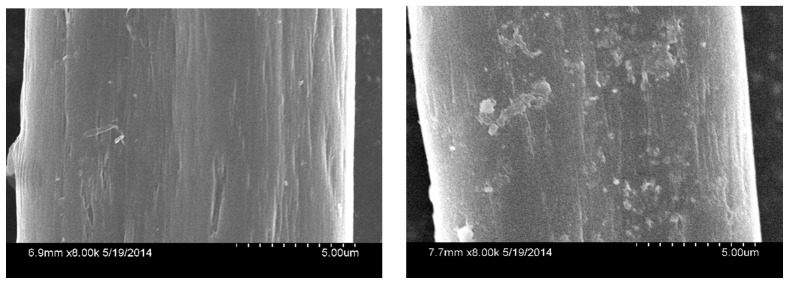
SEM image of unfunctionalized and TNP-functionalized polyester-cotton (65/35) silk.

TNPs were incorporated into polyester and polyester-cotton (65/35) fabrics using the pad-dry-cure process. The quantification of TNP loading was obtained by analyzing total Ti content in nanofabrics using ICP-AES. The TNP loading for polyester-cotton (65/35) nanofabrics was 80 mg/g. The incorporation of TNPs into fabrics was also observed by their SEM images (Figure 2). These results demonstrate that photocatalyst TNPs were successfully incorporated into polyester-cotton nanofabrics for air cleaning while preserving their regular function.

### 2.3. Photocatalytic Degradation of Formaldehyde by Nanofabrics

**Figure 3:**
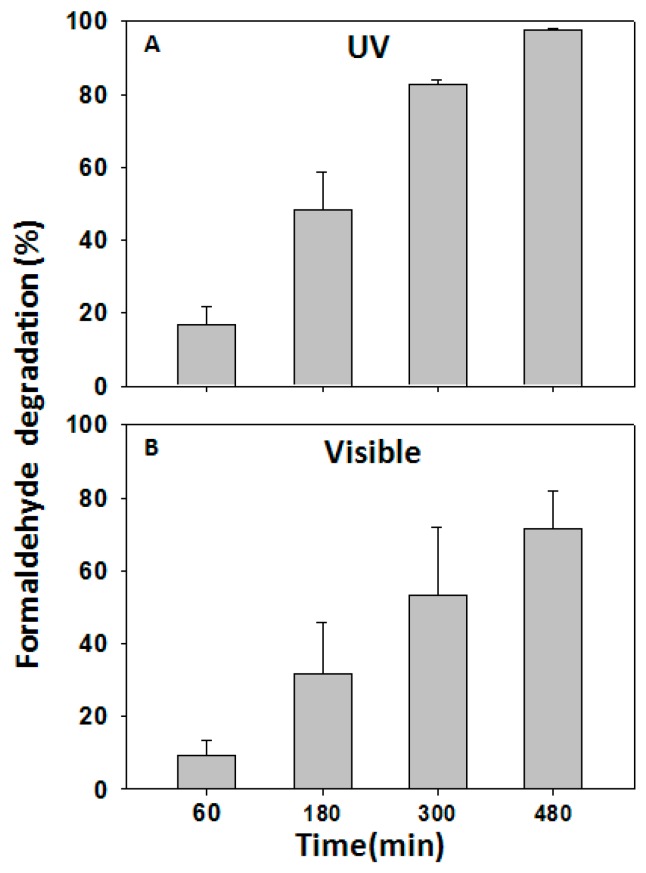
Degradation of formaldehyde by the 65/35 polyester/cotton TNP nanofabrics under UV radiation (**A**) and visible light (**B**). The error bars indicate the standard deviation value (SD) from average values of three independent measurements.

For nanofabrics to be able to clean indoor air, the first priority is to degrade formaldehyde efficiently under visible light irradiation. When nanofabrics were irradiated with visible light in a sealed chamber saturated with formaldehyde (1.5 mg/m^3^), the catalytic degradation of formaldehyde occurred with time. Results showed that after 8 h, 77% of formaldehyde was degraded under the irradiation of visible light (Figure 3B). Although this value was lower than that under the UV irradiation (99%) (Figure 3A), it is very practical and safe for household application under the regular room light. In contrast, the degradation of formaldehyde in the absence of TNP-functionalized polyester-cotton was lower than 20% after 7 h of irradiation without nanofabrics. Since formaldehyde is completely degraded into CO_2_ and H_2_O according to the literature, we believe that this is most likely the case in our reaction chamber. We cannot confirm or exclude the existence of trace amount of other side products because we did not characterize all byproducts.

### 2.4. TNPs Are Firmly Attached to Nanofabrics

To evaluate the possible impact of nanofabrics on indoor environments as reported previously [28], we examined the possible release of TNPs into the air in an indoor environment using a standard fastness to rubbing test (ISO105-X12). Ti content on the nanofabrics before and after dry rubbing was analyzed by ICP-AES. The loss of TNPs after rubbing was near zero for both nanofabrics (Table 2) indicating the stability of nanomaterials on nanofabrics. These results affirmed that these nanofabrics were safe for indoor environments when used as household products. Such stability may be highly related to the specific production processes of nanofabrics. Therefore, standard fastness to rubbing tests should be required for any nanofabric product made by various processes.

**Table 2:** TNP transfer after dry rubbing.

Fabrics Sample	TNP Loading (mg/g) *	TNP Transfer
Polyester nanofabrics	48.7 ± 0.89	1.11%
Polyester nanofabrics After rubbing	48.1 ± 0.33	
Polyester-cotton 65/35 nanofabrics	76.3 ± 0.54	1.14%
Polyester-cotton 65/35 nanofabrics After rubbing	75.4 ± 0.85	

* Values are the means of three independent experiments ± SD.

### 2.5. Toxicity of Free TNPs to Human Cells

Experimental results from the above section indicated that the chance for nanoparticles to be released from nanofabrics was extremely small. However, it could not exclude the possibility that a small amount of TNPs might be released to indoor air, drinking water, food, and eventually the human body. Although an estimated TNP concentration might be very low, we took the precaution of evaluating the safety of TNPs by examining their effects on human cells over a wide range of concentrations up to 220 μg/mL, which is a concentration at which other nanoparticles, such as Ag nanoparticles and carbon nanotubes, show considerable cytotoxicity. Previous studies show that nanoparticle exposure through inhalation or oral or skin contact are eventually absorbed into blood circulation. These nanoparticles are mostly accumulated in the liver or kidneys before excretion. Macrophages in liver, spleen and lungs are the primary cells to internalize these nanoparticles. Therefore, the appropriate cells for safety evaluation are macrophages and cells from the liver and kidneys. With these in mind, we carried out cytotoxicity assays using THP-1 macrophages, HepG2 and HEK293 cells, with PVP-coated silver nanoparticle (PVP-AgNPs) treatment as positive controls.

**Figure 4:**
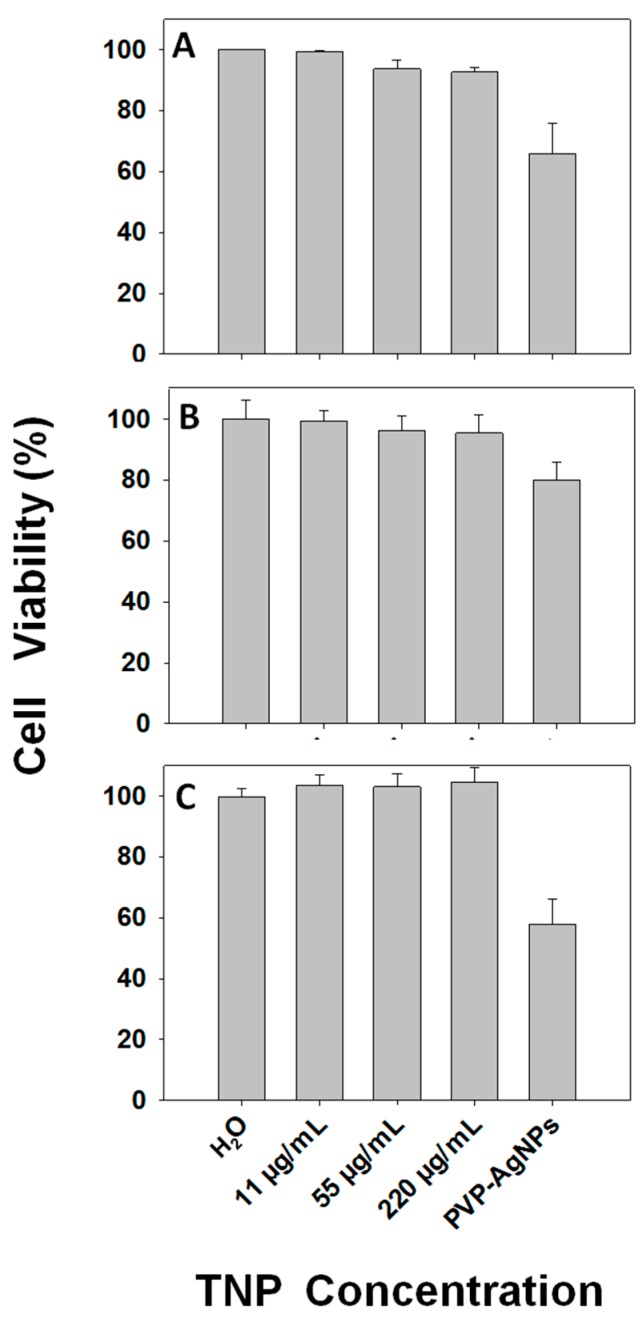
Viability of HEK293 (**A**); HepG2 (**B**); and THP-1 (**C**) treated with TNP at indicated concentrations or silver nanoparticles (PVP-Ag, 50 μg/mL) for 24 h. Values are the means of at least three independent experiments ± SD.

As shown in Figure 4, after a 24 h TNP treatment, cell viability of HEK293, HepG2 and THP-1 macrophages were not affected at all the concentrations tested. In contrast, PVP-AgNPs treatment decreased the cell viability obviously as reported previously [29]. These results indicated that even whenTNPs might be released, they were not toxic to human cells at a wide range of concentrations.

## 3. Experimental Section

### 3.1. Chemicals and Reagents

All chemical reagents were purchased from Sigma-Aldrich (St. Louis, MO, USA) and were used as received. Dulbecco’s Modified Eagle’s Medium and RPMI 1640 medium were purchased from Life Technologies (Grand Island, New York, NY, USA). Fetal bovine serum, 100 U/mL penicillin, and 100 μg/mL streptomycin solution were from HyClone (Logan, UT, USA).

### 3.2. Preparation of TNPs and Nanofabrics

Deionized water, CH_3_CH_2_OH (analytical reagent) and HCl (analytical reagent) were mixed at ambient temperature while alcohol solution of Ti(O–Bu)_4_ was added dropwise by a burette at a rate of 2 drops/s. The mixture was stirred continuously for one hour and then sealed for four days to form TNP hydrosol. Polyester-cotton fabrics (27 mg/cm^2^) were immersed into TNP solution for 5 min. Then fabrics went through cycles of padding-drying (take up 75%–80% of weight, 105 °C, 1.5 min) processes before cured at 170 °C for 1.5 min.

### 3.3. Characterization of TNP and Nanofabrics

The TEM and XRD aqueous solution of TNPs was dispersed by ultrasonication for half an hour. About 10 μL of the solution was added to the copper mesh and dried for 2 h under an infrared lamp. The morphology of the TNPs was observed under a transmission electron microscope (JEM-2100, JEOL, Tokyo, Japan). The crystalline structure of TNPs was analyzed by X-ray diffraction. Using a CuKa radiation source, λ = 0.154 nm, tube pressure 40 KV, electric current 100 Ma, scan range 5–80 °C, a scan speed of 2°/min, and a step size of 0.2°. The hydrodynamic size and the Zeta potential of the naked TNP and TNP-protein complexes were determined according to the method in the previous study [30]. Dynamic diameter of TNP was measured by a dynamic light scattering instrument (Malvern Nano ZS90, Malvern, Worcestershire, UK). Zeta potential of TNPs was measured on a Malvern Nano ZS90 Zetasizer.

### 3.4. Quantification of TNPs in Solution or Nanofabrics

To determine the amount of TNPs in solution or on fabrics, materials were dissolved in concentrated sulfuric acid and the concentration of the Ti was determined by ICP-AES. Each sample was measured three times. Nanofabircs were first burned before the above operations.

### 3.5. Formaldehyde Degradation

Using a previously reported set-up [31], we determined the catalytic degradation of formaldehyde by nanofabrics. Briefly, two pieces of nanofabrics of 0.02 m^2^ (0.04 m^2^ in total) were put on both sides of the irradiation source in the middle of a sealed reactor chamber (324 L). Formaldehyde solution (37%) was added to a heated evaporation plate to make sure all formaldehyde molecules were evaporated and distributed evenly inside the chamber. A formaldehyde flow rate of 1.4 m^3^/min was maintained by a fan inside the chamber to keep formaldehyde molecules in constant movement and in sufficient contact with the nanofabrics. When the equilibrium was reached, the formaldehyde concentration (C_0_, 1.50 mg/m^3^) was determined using a Formaldehyde Quantification instrument (Model POT400, Wan An Di Scientific Co. Limited, Shenzhen, China). Then this was repeated after each irradiation period to determine C. The formaldehyde degradation rate was determined using the equation:

*R* = (C_0_ − C_F_)/C_0_ × 100% (1)

All experiments were repeated at least three times. C_0_ and C_F_ are the concentrations of the formaldehyde in the chamber at the beginning and during its degradation.

### 3.6. Detachment Test

A standard dry rubbing test was carried out following international standard ISO105-X12. Briefly, two pieces of nanofabrics (50 × 140 mm) were fastened in a standard crockmeter (Y571D, Wenzhou, China). A dry rubbing cloth rub against the nanofabrics 20 times along the track with a downward force of 9 ± 0.2 N. Nanofabrics were then removed and burnt. Residues were dissolved in HNO_3_:H_2_SO_4_ (1:1) for digestion in a MARS 6 Classic microwave oven (CEM Corporation, Matthews, NC, USA) for 20 min and then quantitatively analyzed with an Optima 7000DV ICP-AES (Perkin Elmer, Waltham, MA, USA). All experiments were repeated three times.

### 3.7. Cell Culture and Cytotoxicity Assays

The human hepatocellular carcinoma epithelial cell line HepG2 and human embryonic kidney cell line HEK293 were cultured in Dulbecco’s modified eagle’s medium with 10% fetal bovine serum, 100 U/mL penicillin, and 100 μg/mL streptomycin solution. THP-1 cells were cultivated in RPMI 1640 culture medium with 10% fetal bovine serum, 100 U/mL penicillin, and 100 μg/mL streptomycin solution. All cells were grown in a humidified incubator at 37 °C (95% room air, 5% CO_2_). The induction of THP-1 cells into macrophages was triggered by phorbol 12-myristate13-acetate (PMA, Sigma, St. Louis, MO, USA) at a concentration of 50 ng/mL for 48 h [17] in 96-well plate before nanoparticles addition. HepG2 and HEK293 cells (5000 cells/well) were seeded in 96-well plates 24 h before their incubation with nanoparticles. The TNP exposure concentrations were 11, 55, and 220 μg/mL. The percentage of survival cells was determined by CellTiter-Glo assay (Promega Corporation, Madison, WI, USA). All experiments were repeated three times.

## 4. Conclusions

As we face ever-increasing applications of nanotechnology-based products in our daily life, a proper evaluation of their impacts on eco-environment and human health is an imperative task. In this investigation, we carried out such an evaluation on TNP-based nanofabrics, which are effective in photocatalytic degradation of formaldehyde in indoor environments. TNP nanofabrics degrade 77% of formaldehyde in 8 h under irradiation from visible light. Very few free TNPs fall off nanofabrics after a standard rubbing test. Therefore, household usage of such materials would cause minimal effect on indoor environments. Assuming some TNPs are possibly released into indoor environments in the life cycle of the nanofabrics, we found that at a wide range of concentrations, up to 220 μg/mL, TNPs cause no toxicity to macrophages and human liver and kidney cells. Our findings demonstrate the safety profile for nanofabrics and suggest that this should become a necessary requirement for any environment-friendly and safe nanotechnology product. However, there is a possibility that photocatalysis of other organic pollutants may produce some carbonyl intermediate, which may adversely affect air quality [32]. Therefore, this should be taken into consideration when evaluating the net effect of a photocatalytic air purifier.
